# Anti-inflammatory strategies for hepatic encephalopathy: preclinical studies

**DOI:** 10.1055/s-0043-1767819

**Published:** 2023-07-24

**Authors:** Rafaela Pinto Coelho Santos, Eliana Cristina de Brito Toscano, Milene Alvarenga Rachid

**Affiliations:** 1 Universidade Federal de Minas Gerais, Instituto de Ciências Biológicas, Departamento de Patologia Geral, Laboratório de Patologia Celular e Molecular, Belo Horizonte MG, Brazil.; 2 Universidade Federal de Juiz de Fora, Faculdade de Medicina, Departamento de Patologia, Laboratório Integrado de Pesquisa em Patologia, Juiz de Fora MG, Brazil.; 3 Universidade Federal de Juiz e Fora, Faculdade de Medicina, Programa de Pós-Graduação em Saúde, Juiz de Fora MG, Brazil.

**Keywords:** Hepatic Encephalopathy, Neuroinflammatory Diseases, Neuroprotection, Models, Animal, Encefalopatia Hepática, Doenças Neuroinflamatórias, Neuroproteção, Modelos Animais

## Abstract

Hepatic encephalopathy (HE) is a potentially reversible neuropsychiatric syndrome. Often, HE causes cognitive and motor dysfunctions due to an acute or chronic insufficiency of the liver or a shunting between the hepatic portal vein and systemic vasculature. Liver damage induces peripheral changes, such as in the metabolism and peripheral inflammatory responses that trigger exacerbated neuroinflammation. In experimental models, anti-inflammatory strategies have demonstrated neuroprotective effects, leading to a reduction in HE-related cognitive and motor impairments. In this scenario, a growing body of evidence has shown that peripheral and central nervous system inflammation are promising preclinical targets. In this review, we performed an overview of FDA-approved drugs and natural compounds which are used in the treatment of other neurological and nonneurological diseases that have played a neuroprotective role in experimental HE, at least in part, through anti-inflammatory mechanisms. Despite the exciting results from animal models, the available data should be critically interpreted, highlighting the importance of translating the findings for clinical essays.

## Resumo

A encefalopatia hepática (EH) é uma síndrome neuropsiquiátrica potencialmente reversível. Muitas vezes a EH causa disfunções cognitivas e motoras devido à insuficiência do fígado ou por um desvio entre a veia porta hepática e a vasculatura sistêmica. O dano no fígado provoca alterações periféricas, como no metabolismo e nas respostas inflamatórias periféricas, que desencadeiam uma neuroinflamação exacerbada. Em modelos experimentais, estratégias anti-inflamatórias têm demonstrado efeitos neuroprotetores, levando a uma redução dos prejuízos cognitivos e motores relacionados à EH. Neste cenário, evidências crescentes têm mostrado a inflamação periférica e no sistema nervoso central como um promissor alvo pré-clínico. Nesta revisão, abordamos uma visão geral de drogas e compostos naturais aprovados pelo FDA para o uso no tratamento de outras doenças neurológicas e não neurológicas, que tiveram papel neuroprotetor na EH experimental, pelo menos em parte, através de mecanismos anti-inflamatórios. Apesar dos resultados empolgantes em modelos animais, os dados avaliados devem ser criticamente interpretados, destacando a importância da tradução dos achados para ensaios clínicos.

## HEPATIC ENCEPHALOPATHY: AN OVERVIEW

Hepatic encephalopathy (HE) is a common and debilitating complication of acute or chronic liver disease. 1 This condition is a potentially reversible neuropsychiatric syndrome that involves cognitive and motor dysfunctions due to an insufficiency of the liver or shunting between the portal and systemic vasculature, leading to a failure of blood detoxification in the portal circulation. 2 Approximately 30 to 70% of patients with cirrhosis are affected by overt or minimal HE. 3 Accurate data on the prevalence and incidence of this disease are missing, since minimal HE is underdiagnosed. 4 In addition to the impact on the health and quality of life of patients, this condition represents an increasing burden on the health care system. 2 Yearly, the direct costs related to HE ranged between US$ 5,370 and US$ 50,120 per patient worldwide. 3 According to Stepanova et al. (2012), the average cost increased from US$ 46,663 to US$ 63,108 between 2002 and 2009 in the United States. They also showed that the percentage of patients with major and extreme HE severity increased during this period. 5 In a multivariate analysis, the severity of the condition was one of the most important predictors of cost, charge, and length of hospitalization. Patients with a history of HE have a higher risk for relapse, and studies show that it is the main cause of cirrhosis-related rehospitalization. 6 7 8

The severity of HE often impairs the clinical treatment and survival of patients with cirrhosis. 5 9 Bustamante et al. (1999) demonstrated a reduced life expectancy of cirrhotic patients with this condition. The cumulative survival rates at 1 and 3 years after the presentation of the first episode of acute HE were 42 and 23%, respectively. They also showed that the survival probability of patients with HE was lower than expected for liver transplant recipients, regardless of the prognosis. 9 In addition, patients with severe HE awaiting liver transplantation had a 66% higher risk of death than patients without. These data indicate that HE is a strong predictor of mortality among cirrhotic patients. However, this neuropsychiatric syndrome is underestimated in the priority criteria for liver transplantation. 10 Cirrhosis-related HE impairs the ability to perform daily activities and requires more complex therapeutic management. 11 Moreover, this condition and the consequent cognitive dysfunction are associated with worse employment, income, and caregiver burden, impacting the familiar background and medical adherence. 12

The classification of HE is based on the clinical symptoms and etiology. The West Heaven Criteria (WHC) are commonly used for grading the disease. According to WHC, there are five categories for HE: minimal and grades I, II, III, and IV. Minimal HE comprises alterations in psychomotor and executive functions or abnormal neurophysiological tests without clinical manifestations. Grade I comprises patients disorientated to space and time but presenting cognitive or behavioral changes in comparison to their clinical background or reported by the caregiver; in this phase, anxiety and lack of awareness and attention are recurrent symptoms. Grade II comprises patients disorientated for time, exhibiting lethargy and unappropriated behavior. Grade III includes response subjects who are disoriented for space, somnolent, confused, and presenting bizarre behavior. In Grade IV, patients are in a coma and completely unresponsive. 13 14

Despite the excellent reliability in the classification of grades II through IV, the early phase grading is complex, as the symptoms can be mild and often overlap. Accordingly, the European Association for the Study of the Liver (EASL) and the American Association for the Study of Liver Diseases (AASLD) guidelines divided HE into covert (minimal and grade I) and overt (grades II-IV). 13 15 Although covert HE is characterized by mild clinical manifestations, it is associated with falls, incompetent driving, fatigue, and impairments in selective attention and working memory. 16 17 Overt HE exhibits clinically apparent motor abnormalities, such as hyperreflexes, hypertonia, asterixis, bradykinesias, rigidity, tremors, and ataxia. 18 Regarding its etiology, this disease can be classified as type A when prompted by acute liver failure, type B as a consequence of portosystemic shunts in the absence of liver dysfunction, and type C when there is liver cirrhosis and/or portosystemic bypass. 13

The treatment of HE depends on its severity and etiology. It aims to improve quality of life and prevent brain damage, illness recurrence, hospitalization, and death. 18 According to the AASLD/EALD guidelines, four axis should be considered in the decisions on clinical therapy: underlying diseases, HE severity, time-course, and whether the episode is precipitated or spontaneous. 13 The goal of first-line therapy is to reduce intestinal absorption and/or increase the metabolism of ammonia. When the patient is refractory to standard treatments, large portosystemic shunts are considered. End-stage liver disease can be an indication for transplantation. 17

Hyperammonemia plays an important role in the pathophysiology of HE, and the plasma levels of ammonia can also be increased in the absence of symptoms. 19 Furthermore, the correlation between peripheral ammonia levels and the severity of HE-related clinical symptoms is not linear or exponential. 20 In line with this, additional therapeutic targets should be investigated. A growing body of evidence has suggested the involvement of both systemic and central nervous system (CNS) inflammation in the pathogenesis of this condition. Anti-inflammatory strategies have demonstrated neuroprotective effects, reducing cognitive decline and motor activity impairments in experimental HE. 21 22 23 24 These data indicate that inflammation is a promising preclinical target and demonstrate the importance of translating the findings for clinical essays.

## HYPERAMMONEMIA AND INFLAMMATION IN HEPATIC ENCEPHALOPATHY

The mechanisms involved in the etiopathogenesis of hepatic encephalopathy (HE) are not completely understood. Hyperammonemia and inflammation seem to play an important role in the pathophysiology of HE. 25 26 27 28 Ammonia, a toxic molecule, is turned into urea by the liver and is excreted by the kidneys. 28 During acute or chronic liver failure, the urea cycle is impaired, leading to increased levels of serum ammonia, well known as hyperammonemia. 29 In the CNS, ammonia neutralization occurs through the conjugation of this molecule with glutamate by the glutamine synthase (GS) enzyme. This metabolic reaction releases glutamine and occurs in astrocytes. 28 Glutamine accumulation impairs astrocytic function and increases osmotic pressure, which leads to water uptake, prompting swelling of astrocytes and brain edema. 27 30 In addition to the imbalance in osmotic pressure, hyperammonemia may also influence the inflammatory response and neurotransmitter release. 27 29 31

Systemic inflammatory response syndrome (SIRS) associated with liver disease is common among patients with acute liver failure (ALF). In this scenario, increased expression of proinflammatory cytokines (“cytokine storm”), mainly tumor necrosis factor α (TNF-α), interleukin (IL)-1β, and IL-6, is observed in human and experimental ALF. 27 31 32 Peripheral inflammation is associated with mortality risk and the development of HE. Moreover, chronic cirrhotic patients are usually immunosuppressed, what contributes to infection, sepsis, and exacerbated inflammatory responses. 33

A growing body of evidence has suggested the important role of peripheral and central inflammation in HE pathophysiology. 27 29 Proinflammatory mediators, such as TNF-α, IL-1β and IL-6, are released into the bloodstream and may cross the blood–brain barrier, leading to neuroinflammation and oxidative stress. Peripheral TNF-α induces oxide nitric (NO) formation, which activates the nuclear factor-kappa B (NF-kB) intracellular signaling cascade, leading to increased systemic TNF-α mRNA expression in a cyclical process. 30 Moreover, peripheral leukocytes prompt the activation of astrocytes and microglia. 32 34 35 Gliosis contributes to the maintenance of the increased expression of proinflammatory cytokines and oxidative stress in the CNS. 32 34 35 It is worth mentioning that activated microglia play an important role in inflammatory responses and the restoration of homeostasis. However, when chronically activated, this cell assumes a nonresolving role. 32

Microglia may have dual phenotypes, proinflammatory M1 and anti-inflammatory M2, which have been related to both detrimental and beneficial effects in neuroinflammation-related diseases. Previous work reported that treatment with sulforaphane promoted polarization of microglia to a neuroprotective M2 phenotype in the cerebellum of hyperammonemic rats, increasing M2 markers (IL-4, IL-10, Arg 1, and YM-1) and reducing M1 markers (IL-1β). 36 The regulation of microglial polarization can be a potential therapeutic approach to suppress neuroinflammation in neurodegenerative diseases. 37 38 Some authors have described that microglial polarization alters microglia-astrocyte immune interactions in Alzheimer disease. 39 Activated microglia can induce A1 astrocytes by secreting IL-1 and TNF. These astrocytes do not promote neuronal survival, synaptogenesis, outgrowth, or phagocytosis and, instead, induce the death of neurons and oligodendrocytes in neurodegenerative disorders. 40 41 The progression of HE has been associated with increased levels of proinflammatory cytokines and microglial activation in both human and experimental models of liver failure. 32

**Figure 1:**
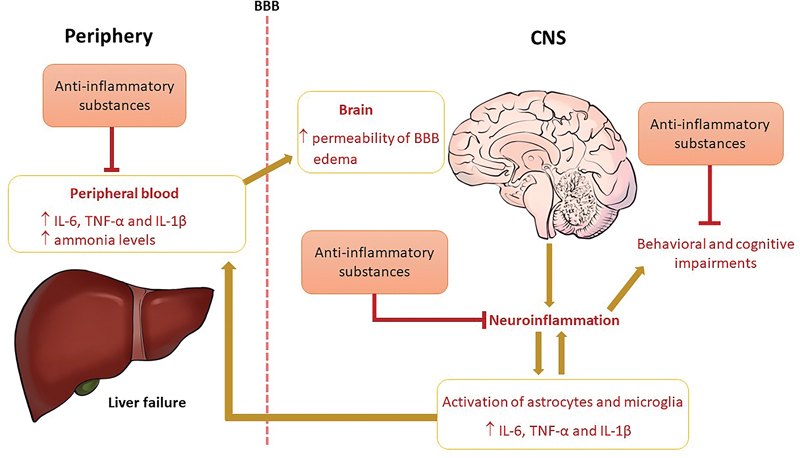
Schematic figure representing the mechanism of development of HE and substances with anti-inflammatory effects Liver failure increases peripheral inflammation and ammonia levels. In this scenario, ammonia and systemic mediators of inflammation can cross the blood brain barrier (BBB), activating microglia and astrocytes and leading to exacerbated neuroinflammation. Glial activation and increased levels of inflammatory mediators induce behavioral and cognitive impairments. Substances with anti-inflammatory effects reduced serum levels of inflammatory mediators and ammonia. In addition, they can decrease brain levels of tumor necrosis factor α (TNF-α), interleukin (IL)-6, and IL-1β levels, as well as inhibit activation of microglia and astrocytes. Anti-inflammatory effects in both the periphery and central nervous system (CNS) can improve behavioral changes and ameliorate the clinical outcomes of animals with HE.

Hyperammonemia and neuroinflammation can act synergistically in the progression of HE ( Figure 1 ). 25 27 30 42 Hyperammonemia promotes oxidative and nitrosative stress (ONS), cellular senescence, and glial activation, inducing neuroinflammation. 30 Intracellular pathways, such as NF-kB, are involved in ammonia neurotoxicity and the neuroinflammation pathway. In astrocytic culture, exposure to ammonia and IL-1β led to NF-kB activation and oxidative stress through increased inducible nitric oxide synthase (iNOS) and hemoxygenase-1 (HO-1) expression. 30 Hyperammonemia can also induce microglial activation directly. 30 32 Conversely, cirrhotic patients can develop advanced HE with infection and systemic inflammation. 43 There are divergences in the direct correlation between ammonia concentration and the severity of HE in patients, suggesting that other factors might contribute to the development of cognitive deficits. 44 As mentioned, a growing body of evidence has proposed the role of inflammation, whether caused by infection or as a consequence of ALF itself, as a central mechanism determining the severity, progression, and outcome of HE. 44 In this context, liver failure induces exacerbated peripheral inflammation that increases the permeability of the blood-brain barrier (BBB), leading to neuroinflammation ( Figure 1 ). Neuroinflammation plays an important role in HE-related behavioral, cognitive, and motor disorders. Anti-inflammatory strategies have demonstrated neuroprotective effects, reducing cognitive decline and motor activity impairments in experimental HE. 21 22 23 24 In line with this, the investigation of the inflammatory mechanisms underlying experimental HE represents a promising path for the elucidation of the pathophysiology of human HE, as well as for the development of novel therapeutic targets. 35

### Impacts of HE-induced neuroinflammation on behavioral, cognitive, and motor functions

Recent evidence has shown an association between glial activation and neuropsychiatric disorders such as depression, anxiety, bipolar disorder, autism spectrum disease (ASD) and neurodegenerative disorders such as Alzheimer disease (AD) and Parkinson disease (PD). In human postmortem studies, patients with bipolar disorder showed higher expression of IL-1β, IL-1β receptor, NF-kB subunits, iNOS, and astromicrogliosis in the frontal cortex. Systemic diseases involving peripheral inflammation (such as, obesity, autoimmune diseases, and infection) are also related to neuroinflammatory mechanisms, cognitive impairment, and depressive- and anxiety-like behavior. 32 35

Astrocytes and microglia are essential to CNS homeostasis. These cells act in the defense against pathogens, elimination of toxic substances, neurodevelopment, synaptic plasticity, and physiological neurodegeneration. In addition, since glial cells express neurotransmitter receptors and transporters, acting in the signaling and reuptake of neurotransmitters, they play a role in cognition, motor function, and behavior modulation. 45 Glia cells participate in the signaling and reuptake of glutamate and γ-aminobutyric acid (GABA). 45 This acid is the main inhibitory neurotransmitter in the CNS. Changes in the GABAergic pathway in the cerebellum cause disturbances in motor function. The reduction in neuroinflammation is related to GABAergic system normalization in the cerebellum and improvements in motor coordination and activity. Glutamate, the main excitatory neurotransmitter in the CNS, is involved in learning and memory processes. The HE-related neuroinflammation induces cell depolarization and ion imbalance in astrocytes and microglia, leading to changes in both GABAergic and glutamatergic pathways and, consequently, to impairment in cognitive and motor functions. 46 47

Experimental models of HE are essential for a better understanding of the mechanisms underlying the development, progression, and possible treatments of this disease. Experimental HE is usually induced using hepatotoxic drugs, a hyperammonemic diet, portacaval shunts (PCSs), and bile duct ligation (BDL). 31 These models prompt increased serum levels of alanine aminotransferase (ALT), aspartate aminotransferase (AST), and ammonia, as well as systemic inflammatory mediators. Experimental models are able to mimic HE-induced psychomotor and cognitive dysfunction observed in patients and demonstrate HE-related inflammation in the hippocampus and cerebellum. 48

Administration of hepatotoxic drugs is considered a satisfactory model of ALF-related HE (type A), while surgical models (such as bile duct ligation) are widely used for the reproduction of HE due to chronic liver failure (type B). To date, there has been no satisfactory model mimicking HE Type C. 31 49 Regarding HE-induced inflammatory mechanisms, comparative evidence on the suitability of models is still missing. However, a growing body of evidence has demonstrated the administration of thioacetamide (TAA) as an inducer of acute liver injury and HE through the induction of oxidative stress and systemic inflammation, similar to that observed in acute HE patients. Both the plasma and brain levels of IL-1β, IL-6, and TNF-α were higher in TAA-treated rats than in control rats. 50 51 52 In mice, Oliveira et al. showed elevated levels of IFN-γ and CCL2 in the prefrontal cortex and of TNF, IL-6, IL-12, and CCL2 in the hippocampus of TAA-treated animals. 53 Alongside these data, TAA intoxication was associated with microglial and astroglial activation. 54 Briefly, acute injection of TAA leads to liver injury, impairing the elimination of blood toxins, such as ammonia and lipopolysaccharides. These agents produce systemic inflammation through the activation of circulatory monocytes and neutrophils. The ammonia and other toxic agents then reach the brain and lead to neuroinflammation and neuronal loss. Therefore, the TAA model in rodents has been introduced as a valid model for studying the crosstalk in HE between peripheral and neuroinflammation. 31 54

## NEUROINFLAMMATION AS A THERAPEUTIC TARGET IN HE

As a multifactorial disease, HE is complex, and its treatment faces several gaps. Pharmacological strategies are based on the reduction of the absorption and/or improvement of ammonia metabolism, control of intestinal microbiota and endotoxemia. There is an intimate communication between gut microbiota and liver in the metabolism and elimination of substances, such as ammonia. Gut microbiota degrades proteins into ammonia, which is neutralized into urea in the liver. During liver failure, this cycle is impaired, leading to hyperammonemia. In addition, some bacteria can produce compounds that increase the permeability of gut barrier function, inducing endotoxemia. 55 Imbalance between beneficial, autochthonous taxa, and pathogenic microbiota in the stool and colonic mucosal is associated with systemic inflammation, and cognitive impairment in patients with mild and overt HE. In turn, the effect of the inflammatory milieu and toxins on the brain promotes increased activation of glial cells and exacerbated neuroinflammation. 56

The main drugs used are nonabsorbable oligosaccharide lactulose, L-ornithine L-aspartate (LOLA), acetyl L-carnitine, zinc supplementation, and poorly absorbable antibiotics, such as rifaximin. The goal of this management is to reduce the absorption of ammonia from the intestine or increase ammonia metabolism. 17 These drugs promote partial improvement of the symptoms and reduce HE relapses in some patients. However, collateral effects (diarrhea and fever), low efficacy, and low tolerability impair medication adherence. Another concern is about individuals with severe HE who are not responsive to the recommended medication management, which reinforces the emergent necessity to study new therapeutic targets. 2

As mentioned previously, in addition to hyperammonemia, inflammation is an important mechanism involved in HE pathogenesis. Recent evidence has demonstrated that experimental drugs with anti-inflammatory effects can ameliorate cognitive and motor function, as well as depressive- and anxiety-like behaviors, supporting the role of neuroinflammation in HE pathophysiology ( Figure 1 ). In this article, we reviewed some exciting data on the beneficial preclinical role of drugs with anti-inflammatory effects in HE.

### Drugs already approved for clinical use as potential therapeutic targets in HE

#### Antibiotics and anti-inflammatory drugs

Drugs currently used clinically in the treatment of inflammatory, autoimmune, and neurodegenerative diseases have been studied as potential therapeutic targets in preclinical HE.

In this scenario, pretreatment with minocycline, a tetracycline antibiotic, prevented microglial activation in the frontal cortex, thalamus, and hippocampus of rats submitted to portocaval anastomosis associated with hepatic arterial ligation. Minocycline attenuated inflammation, decreasing the brain levels of IL-1β, IL-6, and TNF and the mRNA expression of these proteins in the cerebral cortex. Moreover, minocycline retarded the progression of neurological symptoms underlying HE. 57

In another ALF experimental model induced by galactosamine injections, rats pretreated with minocycline or dexamethasone (a corticosteroid used clinically) exhibited improved liver parameters and decreased oxidative and nitrosative stress. Reductions in the levels of IL-1β, IL-6 and TNF and an increase in IL-10 were also observed. However, the beneficial effects of minocycline and dexamethasone on motor activity and mortality were not clear when the pretreated ALF group was compared with the nonpretreated group. 58

Ibuprofen, a nonsteroidal anti-inflammatory drug (NSAID), has also been evaluated in preclinical essays of HE. The intraperitoneal administration of ibuprofen in PCS rats improved learning ability 59 60 and motor activity. 60 61 Ibuprofen restored the brain levels of prolyl oligopeptidase (PREP), decreasing the activity of iNOS and COX. 59 60 61 62 Furthermore, PREP has been implicated in neuroinflammatory events in neurodegenerative diseases and seems to ameliorate the severity of neuroinflammation in patients with cirrhosis. 62

#### Monoclonal antibodies

Etanercept (ETA), a TNF-neutralizing drug used to treat rheumatoid arthritis, attenuated liver and brain damage in an azoxymethane (AOM)-induced acute liver failure model. This drug was able to decrease the levels of hepatic and peripheral inflammation parameters, such as the plasma levels of ALT, AST, ammonium, TNF, and IL-6. Moreover, ETA reduced microglial activation and increased the brain concentration of glutathione, an important antioxidant. 63

Intravenous injections of infliximab, used in the treatment of Alzheimer disease, modulated peripheral inflammation, reducing IL-6 and prostaglandin (PGE2) levels and increasing the expression of IL-10 in male Wistar rats submitted to PCS. 22 64 In addition, the drug improved the performance of the treated rats in tests of memory, learning ability, and motor coordination. The anti-inflammatory effects were associated with modulation of the glutamatergic and GABAergic pathways in the hippocampus 22 and cerebellum, 64 respectively.

#### Angiotensin II receptor antagonists

A chronic thioacetamide (TAA) intoxication-induced chronic liver failure (CLF) model in rats led to inflammatory infiltrate into the liver and increased peripheral levels of ALT, γ-glutamyl transpeptidase (GGT), ammonia, malondialdehyde (MDA), and TNF-α mRNA. Fibrosis of portal areas, collagen deposition, and portal vessel congestion were also observed. Treatment with losartan or candesartan, angiotensin II receptor antagonists, rescued all these parameters. In addition, candesartan improved motor function in rats with CLF. 65 In chronic liver diseases, transforming growth factor β1 (TGF-β1) contributes to the progression of fibrosis. This mediator is upregulated by the renin–angiotensin system (RAS) through the angiotensin II (Ang II) pathway. Since losartan and candesartan are angiotensin receptor blockers (ARBs), they can modulate the deleterious effects induced by this pathway. 65

#### Phosphodiesterase-5 (PD-5) selective inhibitors

Sildenafil improved motor coordination 66 and enhanced memory, spatial learning, 23 and learning ability in rats with HE. 67 Rats treated with this drug exhibited decreased microglial activation and levels of IL-1β and TNF in the cerebellum 66 and hippocampus 23 compared with nontreated HE rats. Alongside this, the administration of sildenafil was associated with increased levels of anti-inflammatory cytokines, such as IL-4, in the cerebellum. 66 In addition, sildenafil seems to inhibit the GABA pathway and stimulate the glutamate pathway in the cerebellum and hippocampus, respectively. These changes can explain, at least in part, the improvement of motor coordination, spatial learning, and memory in treated rats. 23 66

Another efficient PDE5 inhibitor is tadalafil. In the TAA-induced HE model, oral treatment with this drug improved learning, memory, and neuroplasticity in mice through the increase in brain-derived neurotrophic factor (BDNF) and synaptophysin levels. 68 The rescue of cognitive functions can be explained, at least in part, by the tadalafil-induced reduction in astrocytic and microglial activation, as well as decreased TNF, IL-1β, and IL-6 levels in the cerebral cortex and hippocampus. 68

#### Neuromodulators

Bicuculline is a neuromodulator that antagonizes the GABA _A_ receptor. Recent evidence suggests that the drug may mitigate HE in hyperammonemic rats. Bicuculline reduced astrocyte activation and IL-1β levels, modulated glutamate receptor mRNA expression in the hippocampus, positively affected the learning index and memory, and decreased anxiety-like behavior in hyperammonemic rats. 69

Table 1 summarizes the anti-inflammatory effects and the modulation of behavior and cognition related to the aforementioned drugs, as well as the experimental model used.

**Table 1:** Antibiotics, anti-inflammatories, monoclonal antibodies, angiotensin receptors blockers (ARBs), phosphodiesterase-5 (PDE5) inhibitors, and neuromodulators

**Drug**	**Action**	**HE Model**	**Peripheral parameters and systemic inflammation**	**Central parameters and neuroinflammation**	**Behavioral, cognitive, and motor parameters**	**Reference**
Minocycline i.p. ^A, B^	Antibiotic	Male Sprague–Dawley rats. ALF: Portocaval anastomosis + hepatic arterial ligation Male Albino rats: Galactosamine-induced ALF	↓ AST, ALT ↓ Ammonia	↓ Microglial activation ^A^ ↑ IL-10 ^B^ ↓ IL-1β, IL-6, TNF-α ^B^ ↓ IL-1β, IL-6, TNF-α _mRNA_^A^ ↓ Nitrite/Nitrate ^B^ ↓ iNOS, heme oxygenase-1 gene expression ^B^ ↓ Brain water ^B^ ↓ Brain edema ^A^	+ Time to loss of righting and corneal reflex	**A** : Jiang et al., 2009 57 **B** : Gamal et al., 2013 58
Dexamethasone i.p.	Corticosteroid	Male Albino rats: Galactosamine-induced ALF	↓ AST, ALT ↓ Ammonia	↑ IL-10 ↓ IL-1β, IL-6, TNF-α ↓ Nitrite/Nitrate ↓ iNOS, heme oxygenase-1 NA: Brain water		Gamal et al., 2013 58
Etanercept (ETA) i.p.	Anti TNF- α	Male C57/BL6 mice AOM-induced AFL	↓ AST, ALT ↓ Ammonia, TNF-α, IL-6, ↑ GSH + Liver histology: ↓ necrosis, vacuolization hepatocyte and congestion	↓ Microglia ↓ IL-6 NA: GSH	↑Time to coma	Chastre et al., 2012 63
Infliximab i.v. tail ^A, B^	Anti TNF-α	Male Wistar rats: PCS ^A, B^	NA: Ammonia ^A, B^ ↓ PGE2, IL-6 ^A, B^ ↓ IL-17 ^A^ ↑ IL-10 ^A, B^ NA: IL-4 ^B^	• Cerebellum ^B^ : ↓ Microglia and astrocyte activation ↓ cell expressing TNF- α, IL-1β ↓ [TNF- α, IL-1β] Modulate GABA and GABA transporter in astrocyte and neuron • Hippocampus ^A^ : ↓ Microglia activation ↓ Neuron expressing TNF- α NA: cell expressing IL-1β ↓ TNF-α _mRNA_ ↓ [TNF-α, IL-1β] Modulate Glu receptors in brain regions	+ Spatial memory ^A^ + Learning ability ^B^ + Motor coordination ^B^	**A** : Dadsetan et al., 2016a 22 **B** : Dadsetan et al., 2016b 64
Ibuprofen i.p. ^A, B, C^	NSAID	Male Wistar rats ^A, B, C, D^ : PCS ^A, B, C^ BDL ^D^ Hyperammonemic diet ^D^	NA: Ammonia ^B^	• Cerebral cortex: Modulate Glu pathways ^B^ NA: [TNF- α, IL-6] ^B^ ↓ iNOS and COX activity ^B^ • Striatum: ↓ PREP activity ^A^ • SNr: Modulate Glu and Glu transporters ^C^ • Cerebellum: NA: iNOS ^D^ ↓ MHCII ^D^ NA: Ammonia ^D^	+ Learning ability ^B, D^ + Cognitive function ^D^ + Motor activity ^C, D^	**A** : Tenorio-Laranga et al., 2015 62 **B** : Cauli et al., 2007 59 **C** : Cauli et al., 2009 61 **D** : Rodrigo et al., 2010 60
**Drug**	**Action**	**HE Model**	**Peripheral parameters and systemic inflammation**	**Central parameters and neuroinflammation**	**Behavioral, cognitive, and motor parameters**	**Reference**
Losartan gavage	ARB	Male Sprangue-Dawley rats: TAA-induce CLF: MHE	↓ Ammonia, ALT, GGT, MDA ↓ TNF- α _mRNA_ ↑ GSH + Liver histology ↓ Liver fibrosis	–	NA: Psycomotor behavior NA: Locomotory activity	Murad et al., 2017 65
Sildenafil drinking water ^A, B, C^	PDE-5 inhibitor	Male Wistar rats ^A, B, C^ : PCS rats ^A, B, C^ MHE ^A, B^ Hyperammonemic ^C^	NA: Ammonia ^A^	• Cerebellum ^A^ : ↓ Microglia and astrocyte ^A^ ↓ [IL-1β; TNF-α] ^A^ ↑ [IL-4] ^A^ Modulate GABA, GABA receptors and transporters ^A^ Modulate Glu pathway ^C^ • Hippocampus ^B^ : ↓ Microglia ↓ [IL-1β; TNF-α; IL-10] Modulate GABA and Glu receptor expression	+ Motor coordination ^A^ + Spatial learning and memory ^B^	**A** : Augusti et al., 2016 66 **B** : Hernandez-Rabaza et al., 2015 23 **C** : Erceg et al., 2005 67
Tadalafil gavage	PDE-5 inhibitor	Male C57/6 mice TAA-induce ALF	↓ NO _serum_	• Cerebral cortex: ↓ Microglial activation and astrocyte ↓ TNF-α, IL-1β, IL-6 ↑ BDNF, synaptophysin • Hippocampus: ↓ Microglial activation and astrocyte ↓ TNF-α, IL-1β, IL-6 Modulate Glu receptors	+ Spatial learning and memory	Franca et al.,2019 68
Bicuculline i.p.	GABA _A_ receptor antagonist	Male Wistar rats: Hyperammonemic	–	• Hippocampus: NA: Microglia activation ↓ Astrocytes activation ↓ Cell expressing IL-1β ↓ [IL-1β] Modulate Glu receptors	+ Learning index + Memory + Exploratory behavior ↓ Anxiety-like behavior	Malaguarnera, et al., 2019 69

**Abbreviations:** ALF, acute liver failure; ALT, alanine aminotransferase; AOM, azoxymethane; ARB, angiotensin receptor blockers; AST, aspartate aminotransferase; BDL, bile duct ligation; BDNF, brain derived neurotrophic factor; CLF, chronic liver failure; COX, cyclooxygenase; GABA, gamma-aminobutyric acid; GGT, γ-glutamyl transpeptidase; Glu, glutamate; GSH, glutathione; HE, hepatic encephalopathy; i.v., intravenous injection; i.p., intraperitoneal injection; IL, interleukin; iNOS, inducible nitric oxide synthase; MDA, malondialdehyde; MHCII, major histocompatibility complex II; MHE, minimal hepatic encephalopathy; NA nonapplicated or nonameliorated; NO, nitric oxide; NSAID, nonsteroidal anti-inflammatory drug; PCS, portacaval shunt; PDE-5, phosphodiesterase- 5 inhibitor; PGE2, prostaglandin; PREP, polyl oligopeptidase; SNr, substantia nigra pars reticulata; TAA, thioacetamide; TNF-α, tumor necrosis factor α. **Notes:** + improves; - reduces; ↓ decrease; ↑ increase; [ ] concentration.

### Natural compounds, hormones, and oral supplements as potential therapeutic targets in HE

#### Natural compounds

Natural compounds, oral supplementation, and hormones have been investigated as potential substances in the treatment of HE. Cannabidiol (CBD) is an antagonist of cannabinoid receptors (CB) 1 and 2. It is extracted from Cannabis sativa and is a nonpsychotic natural compound. Some studies have demonstrated an anti-inflammatory action of this substance. 70 Intraperitoneal injection of CBD reduced behavioral changes and increased neurological scores, 71 memory performance, 72 and motor function in mice subjected to BDL. 71 72 73 These neuroprotective effects can be explained, at least in part, by the CBD-related increased hippocampal concentration of BDNF 72 73 and the decreased TNF-α levels, 72 TNF-α receptor 1 (TNFRSF1) expression, 73 and astrogliosis in the brains of BDL mice. 71 Additionally, CBD showed a beneficial effect on liver function, reducing the serum levels of AST, ALT, ammonia, and bilirubin. 71

The mixed extracts of Rheum undulatum and Glycyrriza uralensis (RG) are widely used in traditional oriental medicine, showing anti-inflammatory effects. In the experimental murine model of chronic hepatic cirrhosis induced by carbon tetrachloride (CCl _4_ ), RG extract minimized hepatic damage and decreased the necrosis, and inflammatory infiltrate in the liver. Moreover, the drug preserved BBB permeability by reducing metalloproteinase 9 (MMP9) and increasing claudin-5 expression. In the CNS, RG extract reduced neuroinflammation and decreased TGF-β, IL-1β, and astrogliosis, which was associated with improved motor activity in cirrhotic mice. 74

Sulforaphane is a natural compound with antioxidant, anti-inflammatory, and neuroprotective effects. In rats submitted to a hyperammonemic diet that received sulforaphane by the intraperitoneal route, there was a reduction in astrogliosis and microglial polarization to the M2 phenotype (anti-inflammation microglial profile) in the hippocampus 75 and cerebellum. 36 Additionally, sulforaphane enhanced the levels of anti-inflammatory cytokines, such as IL-10 and IL-4, in the cerebellum. 36 It also affected the modulation of GABAergic and glutamatergic pathways in the hippocampus, 75 and the GABAergic pathway in the cerebellum. 36 Furthermore, sulforaphane administration promoted better performance in tests of spatial learning, 75 learning ability, and motor coordination in treated animals than in nontreated ones. 36

Another potential therapeutic target reported in HE is fish oil supplementation. Brain cellular functions are significantly influenced by omega-3 polyunsaturated fatty acids, which are essential components of cell membrane phospholipids. Chronic fish oil (FO) supplementation, which is rich in omega-3 polyunsaturated fatty acids, ameliorated spatial memory and oxidative stress in Wistar rats with HE induced by TAA intoxication. 76 Polyunsaturated fatty acids are substrates for specialized pro-resolving mediators, such as lipoxins, resolvins, and protectins. These lipid mediators dampen inflammation and promote host defense without causing immunosuppression. 77 In addition, they have a hepatoprotective role by reducing liver inflammation, fibrosis, and steatosis. 78 Resolvin E1 mitigates the progression of liver fibrosis in Sprague–Dawley rats by attenuating fibrogenesis and restricting proliferation. 79

#### Hormones and vitamins

Aghei et al. (2014) and Golshani et al. (2019) showed positive effects of erythropoietin (EPO) administration in rats subjected to BDL. It restored hepatic parameters 80 and decreased neurodegeneration and gliosis in both the hippocampus and cerebellum of the BDL-treated group in comparison to the untreated group. 80 This drug also improved motor function, restored fear learning, and ameliorated performance in tests of spatial learning and memory. 81 Several lines of evidence have demonstrated that EPO reduces neuronal apoptosis and modulates neuroinflammation. The antioxidative effects of EPO, decreasing levels of the reactive oxygen species (ROS) and reactive nitrogen species (RNS), inhibit microglial infiltration by preserving the BBB and decreasing microglial activation. 82

Similarly, fibroblast growth factor-21 (FGF-21) had a beneficial effect on neuroprotection, and antioxidant and synaptic plasticity functions. It is a peptide hyperexpressed during the late phases of liver injury and plays a resolutive role in tissue damage. Opoku et al. (2020) showed that intraperitoneal injections of FGF-21 decreased mRNA expression of fibrosis markers (TGF-β1, histone deacetylase 3 (Hdac3), and collagen 1) in the liver. Moreover, this peptide decreased the serum and brain levels of the chemokine C-C motif ligand (CCL5), TNF-α, IL-1β, and IL-6, as well as increased the mRNA expression of IL-10 in the liver and brain, improving the cognitive and neurologic score in mice submitted to TAA-induced ALF. 83

#### Oral supplementation

In BDL animals, taurine oral supplementation was able to rescue coordination and locomotor activity. It seems that this effect was induced, at least in part, through the reduction of ammonia levels along with increased antioxidant activity in the brain. 84

Another substance that seems to have antioxidant, anti-inflammatory, and hepatoprotective effects is Coenzyme Q10 (CoQ10), which can be produced by humans and is found in nuts, fruits, fish, and meat. Askani-Esfahani et al. (2016) demonstrated that intraperitoneal injection of CoQ10 improved liver parameters, reducing centrilobular necrosis, hepatocyte vacuolization, and inflammatory infiltrate in rats submitted to TAA-induced ALF. In addition, treatment with CoQ10 reduced depressive-like behavior and ameliorated locomotor activity in the ALF group. 85

Table 2 summarizes the main anti-inflammatory effects and the modulation of behavior and cognition related to the aforementioned drugs, as well as the experimental model used.

**Table 2:** Natural compounds, hormones, and supplements

**Drug**	**Kind**	**Model and HE type**	**Peripheral parameters and systemic inflammation**	**Central** **parameter and neuroinflammation**	**Behavioral, cognitive, and motor parameters**	**Reference**
Cannabidiol (CBD) i.p. ^A, B, C^	Natural compound	Female Sabra mice ^A, B, C^ : BDL ^A, B^ TAA-induce ALF ^C^	↓ AST, ALT ammonia, bilirubin ^C^ NA: hepatic necrose ^C^	↓ Astrogliosis ^C^ • Hippocampus: ↓ TNFRSF1A _mRNA_^A,B^ ↑ BDNF ^A, B^ NA: COX ^B^	+ Cognitive function ^A, B, C^ + Motor function ^A, B, C^ + Neurological score ^C^	A: Magen et al., 2010 73 B: Megan et al., 2009 72 C: Avraham et al., 2010 71
*Rheum undulatum and Glycyrriza uralensis* (RG) mix extract Drinking water	Herbal medicine: medicine tradicional oriental	Male Balb/C mice CCL _4_ -induced HE	↓ ALT, ammonia + Liver histology: ↓ inflammation, necrosis, vacuolization of hepatocytes	↓ Astrogliosis ↓MMP9 ↑ Claudin 5 ↓ TGF-β1, IL-1β _Mrna_	+ Locomotor activity and distance traveled	Baek et al., 2020 74
Sulforaphane i.p. ^A, B^	Natural compound	Male Wistar rats: Hyperammonemic diet ^A, B^	NA: Ammonia ^A, B^	• Cerebellum ^B^ : NA: Microglia ↑ M2 macrophage ↓ Astrogliosis ↑ IL-10, IL-4 ↓ IL-1β ↓ Cell expression IL-1β Modulate GABA and GABA transporter Modulate Glu pathway • Hippocampus ^A^ : ↓ Microglia and astrocyte activated ↑ M2 macrophage NA: TNF-α, IL-10, IL-4 ↓ IL-6, IL-1β Modulate GABA and Glu receptors	+ Spatial learning ^A^ + Learning ability ^A, B^ + Motor coordination ^B^	A: Hernandez-Rabaza et al., 2016 75 B: Hernandez-Rabaza et al., 2016 36
Erythropoietin (EPO) i.p. ^A, B^	Hormone	Male Wistar rats: BDL ^A, B^	↓ AST ^A,^ ↓ ALT ^B^ NA: Bilirubin ^A, B^ ↑ Albumin ^A^ NA: Albumin ^B^ ↑ Red blood cells ^A, B^ NA: Hemoglobin ^A, B^	Cerebellum and hippocampus: ↓ Microglia and astrocyte ^A^ ↓ neuronal degeneration ^A^	+ Mobility ^B^ + Balance function ^B^ + Spatial learning and memory ^B^ + Fear learning ^B^	A: Golshani et al., 2019 80 B: Aghaei et al., 2015 87
Fibroblast growth fator-21 (FGF-21) i.p.	Growth factor	Male C57/6J mice: TAA-induce ALF	Serum: ↓ CCL5 Liver: ↓ CCL5 ↑ IL-10 _mRNA_ ↓ TNF-α, IL-1β, IL-6 _mRNA_ ↓ TGF-β1, Hdac3, Colagen1 _mRNA_ ↓ Liver fibrosis	↓ CCL5 ↓ [TNF-α, IL-1β, IL-6] _mRNA_ ↑ [IL-10] _mRNA_ Modulate GABA	+ Cognitive and neurological score	Opoku et al., 2020 83
**Drug**	**Kind**	**Model and HE type**	**Peripheral parameters and systemic inflammation**	**Central** **Parameter and neuroinflammation**	**Behavioral, cognitive, and motor parameters**	**Reference**
Taurine Gavage	Amino acid human and supplementation	Male Sprague–Dawley rats: BDL	↓ Ammonia, ALT, AST ↑Albumin	↓ Ammonia ↓ ROS, lipid peroxidation ↑GSH, antioxidant capacity	+ Motor coordination + Locomotory activity	Heidari et al., 2018 84
Fish oil Oral	Natural compounds	Male Wistar rats: TAA-induced ALF		↓SOD activity	+ Spatial memory	Staziaki et al., 2013 76

**Abbreviations:** ALF, acute liver failure; ALT, alanine aminotransferase; AST, aspartate aminotransferase; BDL, bile duct ligation; BDNF, brain-derived neurotrophic factor; CCL _4,_ carbon tetrachloride; CCL5, chemokine C-C motif ligand 5; COX, cyclooxygenase; GABA, γ-aminobutyric acid; Glu, glutamate; GSH, glutathione; Hdac3, histone deacetylase 3; HE, hepatic encephalopathy; i.p., intraperitoneal injection; IL, interleukin; MMP9, metalloproteinase 9; ROS, reactive oxygen species; SOD, superperoxide dismutase; TAA, thioacetamide; TNFRSF1A, TNF-α receptor 1; TGF-β1, transforming growth factor β 1; TNF-α, tumor necrosis factor- α. **Notes:** + improves; - reduces; ↓ decrease; ↑ increase; [ ] concentration; (NA) nonapplicated or nonameliorated.

## CONCLUSIONS AND FUTURE DIRECTIONS

Although multifactorial mechanisms are involved in the pathophysiology of HE, the current treatment is focused on the reduction of intestinal absorption and/or an increase in the metabolism of ammonia. 17 This treatment faces several gaps and a significant level of drug resistance. End-stage liver disease with severe HE is still a condition commonly referred for liver transplantation and presents high levels of mortality. The therapeutic strategies displayed collateral effects and low efficacy and tolerability, leading to low adherence. 2

Experimental models of HE are essential for a better understanding of the mechanisms underlying the development, progression, and possible treatments of this disease. Preclinical studies suggest inflammation as a potential therapeutic target for the treatment of HE. In this review, we considered FDA-approved drugs used in the treatment of other neurological and nonneurological diseases that have played a beneficial role in experimental HE, at least in part, through anti-inflammatory mechanisms. The modulation of the inflammatory response has demonstrated neuroprotective effects, reducing the HE-related cognitive decline and motor impairments, as well as depressive and anxiety-like behaviors, supporting the role of neuroinflammation in HE pathophysiology.

Despite the promising experimental findings, there is a gap in clinical essays approaching anti-inflammatory strategies in HE, highlighting the importance of translational studies in this area. Patients with chronic active hepatitis have significant impairment in drug metabolism. Low doses of anti-inflammatory substances may cause hematologic, cholestatic, dermatologic, and hepatotoxic adverse effects. In addition, a lot of evidence reveals that anti-inflammatory treatments neither halts the histologic progression of chronic severe hepatitis nor significantly alters the long-term prognosis when initiated in the later stage of the disease. 86 Therefore, future clinical studies should address pharmacokinetic issues, such as doses, absorption, clearance, and toxicity related to anti-inflammatory substances, especially in patients with severe HE and immunosuppressed individuals.
